# Adolescent idiopathic scoliosis associated *POC5* mutation impairs cell cycle, cilia length and centrosome protein interactions

**DOI:** 10.1371/journal.pone.0213269

**Published:** 2019-03-07

**Authors:** Amani Hassan, Stefan Parent, Hélène Mathieu, Charlotte Zaouter, Sirinart Molidperee, Edward T. Bagu, Soraya Barchi, Isabelle Villemure, Shunmoogum A. Patten, Florina Moldovan

**Affiliations:** 1 Faculty of Dentistry, Université de Montréal, Montréal, Québec, Canada; 2 CHU Sainte-Justine Research Center, Montréal, Québec, Canada; 3 Department of Basic Biomedical Sciences, Sanford Medical School, University of South Dakota, Vermillion, SD, United States of America; 4 École Polytechnique de Montréal, Montréal, Québec, Canada; 5 INRS–Institut Armand-Frappier, Université du Québec, Laval, Montréal, Québec, Canada; University of Massachusetts Medical School, UNITED STATES

## Abstract

Adolescent Idiopathic Scoliosis (AIS) is a spinal deformity that affects approximately 3 percent of human adolescents. Although the etiology and molecular basis of AIS is unclear, several genes such as *POC5* have been identified as possible causes of the condition. In order to understand the role of *POC5* in the pathogenesis of AIS, we investigated the subcellular localization of POC5 in cilia of cells over-expressing either the wild type (wt) or an AIS-related *POC5* variant *POC5*^*A429V*^. Mutation of POC5 was found to alter its subcellular localization and to induce ciliary retraction. Furthermore, we observed an impaired cell-cycle progression with the accumulation of cells in the S-phase in cells expressing *POC5*^*A429V*^. Using immunoprecipitation coupled to mass spectrometry, we identified specific protein interaction partners of POC5, most of which were components of cilia and cytoskeleton. Several of these interactions were altered upon mutation of POC5. Altogether, our results demonstrate major cellular alterations, disturbances in centrosome protein interactions and cilia retraction in cells expressing an AIS-related POC5 mutation. Our study suggests that defects in centrosomes and cilia may underlie AIS pathogenesis.

## Introduction

At present, the etiology and biological mechanisms that are involved in the pathogenesis of adolescent idiopathic scoliosis (AIS) remain unclear. Several etiologies and pathways such as neuroendocrine, neurological, muscular, biochemical and structural, hormonal, mechanical and genetic have been suggested to contribute to AIS [1–4]. We previously reported that mutations in the centrosomal protein-encoding gene *POC5* are associated with familial idiopathic scoliosis in French Canadian families [5]. The involvement of *POC5* in AIS was further confirmed in a case-control study, where the *POC5* variant (rs6892146) was found to be associated in individuals with AIS [6].

In humans, the *POC5* gene is on chromosome 5q13 and encodes an ubiquitously expressed protein, abundant in the centrioles where it interacts with centrin and inversin [7]. POC5 is essential for assembling the distal half of the centriole and the elongation of the centrioles [7]. It is also involved in cell functions such as cell polarity, division, motility, and forms part of the cell cytoskeleton that is important for cell dynamics [7–9]. The localization of POC5 within photoreceptors is crucial for ciliary connection and retinal function [10].

Cilia are organelles that extend from the cellular surface of most eukaryotic cells [11]. There are two types of cilia, motile and nonmotile cilium, the latter is also known as primary cilium. Motile cilia are composed of a 9+2 axonemal structure with nine outer microtubule doublets surrounding two centrally located singlet microtubules, and additional accessory structures [10]. Primary cilium are found in almost all eukaryotic cells and are characterized by their 9+0 axoneme organization. They sense and transduce environmental signal and are critical for embryonic and postnatal development, as well as for tissue homeostasis in adulthood [12]. Due to their broad tissue distribution, defects in primary cilia will result in to a broad range of ciliopathies characterized by phenotypic variability and clinical features ranging from renal, retinal, hepatic, musculoskeletal and central nervous system defects [13–16]. Cilia abnormalities were recently associated with scoliosis and defects in the central nervous system [17]. For instance, in zebrafish, mutation of the protein-tyrosine kinase-7 was shown to affect the formation and function of motile cilia in the central nervous system [17] suggested that the ciliary abnormalities caused a disturbance in the flow of cerebrospinal fluid (CSF) leading into spinal curvature. Given the roles of centrosomal proteins in ciliogenesis [18], it is very likely that mutations in POC5 would impact cilia function. However, this hypothesis remains to be explored.

In this study, we investigated the impact of mutations in *POC5* on primary cilia and the subsequent implications in the pathogenesis of AIS. We show that an AIS-related mutation in POC5 induce ciliary retraction and impair cell-cycle. We further demonstrate that mutated POC5 loses its ability to interact with proteins that are important for cilia function as well as cytoskeleton organizations.

## Materials and methods

### Ethical considerations

All human tissue samples were collected in accordance with the policies regarding the ethical use of human tissues for research. The protocol used in this study was approved by the Centre hospitalier universitaire Sainte-Justine Ethics Committee (# 3704).

### Cellular localization of POC5

All cells used in this study were cultured in DMEM media (Wisent cat: 319-015-CL) in an eight-well-chamber glass slide (Fisher scientific cat: 354108). HeLa cells were transfected with either Myc tagged wt-*POC5* (Origene cat: RC211731) or *POC5*^*A429V*^, (generated by site directed mutagenesis [5]). Following, a 24 hours period post transfection with lipofectamine (Invitrogen, cat: 11668–019), the cells were processed for immunocytochemistry analysis. Briefly, the cells were fixed on ice using 70% ethanol and 0.2% triton, permeabilized with 0.1% triton in PBS (PBT), and then incubated for one hour at room temperature (RT), with rabbit polyclonal POC5 antibody (abcam cat: ab188330, 1/250) and mouse monoclonal acetylated-α-tubulin antibody (Sigma Aldrich cat: T7451 1/2000) diluted in 2% BSA/PBT. After incubation with the primary antibodies, the cells were washed three times with PBT, incubated for one hour at room temperature with secondary antibodies (1/500) Alexa fluor 488 anti-rabbit (life technologies cat: A11008) and Alexa fluor 555 anti-mouse (Life technologies cat: A21422). Samples were then mounted using prolong gold anti-fade reagent with diamidino-2-phenylindole dye for fixed cells (DAPI) (Life technologies cat: P36931). Confocal fluorescent images were captured using a Zeiss microscope, Z-stack digital imaging was performed in order to reveal more details of the position of POC5 with respect to cilia. The Z-stack technique combines multiple images taken at different focal distances to give a subsequent image with a greater depth of field. Acetylated-α- tubulin (a ciliary marker), POC5 protein and DAPI (the nuclear counterstain) were visualised as red, green, and blue, respectively.

To analyze expression and cellular localization of POC5 at different phases of the cell cycle, HeLa cells were cultured in DMEM Medium (Wisent cat: 319-015-CL) with 10% FBS (Wisent cat: 080–110) and 1% penicillin streptomycin Glutamine (Wisent cat: 50-202-EL) (PSG). When the HeLa cells were confluent, they were serum starved for 24 hours in order to synchronize their cell cycle. Serum starvation was performed by culturing confluent cells in DMEM medium without FBS, supplied with 1% PSG to block in G1 phase. To block cells in S phase, cells were then supplied with DMEM medium with FBS (10%) and 1% PSG for 24 hours. Cells were then examined by double immunofluorescence as described above. Immunofluorescence studies were also performed on normal human osteoblasts and cells carrying *POC5* variant mutation c.C1286T (p.A429V). Tissue samples were collected for mutation analysis of the osteoblasts from patients with scoliosis during surgery. Genomic DNA was extracted from cells using pure link genomic DNA mini kit (cat: k 1820–01). Polymerase chain reaction was performed for exon 10 using primers: Forward: 5’CTTTTCATAAGGTGGGACCT3’ Reverse: 5’TCCGATGCCCTTACCAG3’. Bands corresponding to the correct molecular weight of *POC5* were excised from gel and purified using GenElute Gel extraction kit (Cat: NA1111-1KT). The purified DNA amplicons were then sequenced (University McGill and Génome Québec Innovation Centre, Montréal QC).

### Preparation of nuclear and cytoplasmic extracts

Cells were washed with 4 ml of PBS (1x) and then harvested in PBS by scraping. The lysate was collected in eppendorf tube and spun for 5 minutes in a microcentrifuge at low speed (4000 rpm). Pellet was washed twice with 500 μl of buffer A (Hepes 10mM, KCl 10mM, DTT 0.5mM)(w/o NP-40) and then cell pellet was resuspended in 20 μl of buffer A (w/ NP-40 (1%)) and incubated at 4°C for 10 minutes with rocking. The cell pellet was spun down in microcentrifuge for 2 minutes at max speed. The supernatant is the cytoplasmic extract (C extract) by adding 55 μl of buffer C and stored at –80°C. The nuclear extract was resuspended in 55 μl of buffer C (Hepes 20 mM, glycerol 20%, KCl 500 mM, EDTA 0.2 mM, PMSF 0.5 mM, DTT 0.5 mM and MgCl2 1.5mM); and incubated at 4°C for 15 minutes with rocking. The nuclei were then spun in microcentrifuge for 5–10 minutes at max speed. At this stage, the supernatant is the nuclear extract (N extract).

### Immunoprecipitation coupled to mass spectrometry (IP–MS), co-Immunoprecipitation and Western blot

To identify specific protein-protein interaction partners of POC5, immunoprecipitation coupled to mass spectrometry, co-immunoprecipitation, and Western Blot analysis was performed on HEK293 cells transfected with wt or *POC5*^*A429V*^ expressing vectors. HEK293 cells were cultured in DMEM medium (Wisent cat: 319-015-CL) with 10% FBS and 1% PSG. When confluent, cells were transfected using lipofectamine 2000 (Invitrogen cat: 11668027) with control (mock-myc empty vector), Myc tagged wt or *POC5*^*A429V*^ expressing vectors. For transfection, cells were maintained in antibiotic free medium overnight. On the second day, cells were transfected based on the lipofectamine protocol provided by the manufacturer (Invitrogen). For protein extraction, cells were lysed in IP lysis buffer (Pierce Thermoscientific, cat: 87787) supplied with 1 x protease inhibitor cocktail (Roche cat: 04693116001). Lysate was then centrifuged for 15 minutes at 8000 rpm. Supernatant was collected for protein quantification by Bradford (Bradford Biorad cat: 500–0006). For immunoprecipitation, 3000 μg of proteins were immunoprecipitated with 2.5 μg/ml of anti-myc antibody (Origene, cat: TA15021) overnight (ON) at 4°C. Magnetic beads (Biorad cat: 161–4023) were washed with PBS and blocked using 2% BSA/PBS (BSA from Sigma-Aldrich cat: A7906) ON at 4°C. On the second day, beads were washed 3 x with PBS and then incubated with protein lysate and antibody complex at 4°C ON. On the third day, the flow through was collected and the beads were heated for 10 minutes in laemmeli 2 x (biorad cat: 161–0737) at 55°C. Protein samples and dual plus molecular weight ladders (Bio-rad cat: 161–0374) were separated by SDS-PAGE approximately 90 minutes at 100 Volts in running buffer (25 mM Tris base, 192 mM glycine, 1% SDS, pH 8.3). The gel was stained with Coommasie blue (Biorad cat G-250 #1610406) for 3 hours. After staining the gel with Coomassie blue, the bands corresponding to the molecular size of POC5 (63 kDa) were excised and analyzed by mass spectroscopy. The gels were subjected to trypsin digestion then an aliquot of the tryptic digest (prepared in 5% acetonitrile/ 0.1% trifluoroacetic acid in water) was analyzed by LC-MS on an LTQ-Orbitrap mass spectrometer system (ThermoElectron) coupled to a Dionex 3000 nano-LC system (Camberley). Mass spectroscopy analysis was evaluated using scaffold software [19]. Mass Spectroscopy was performed at the Mass spectroscopy plateform at the Institute of Research in Immunology and Cancer (IRIC).

In parallel, an aliquot of protein samples was transferred to nitrocellulose membranes (Bio-Rad cat: 9004700) and ran 90 min at 90 V and 250 mA. The total proteins on membranes were detected with Ponceau S staining (Sigma cat: P3504). Membranes were blocked with 20% non-fat milk (Santa-Cruz cat: Sc2324) in PBST (10 mM phosphate, 137 mM NaCl, 2.7 mM KCl, containing 0.05% Tween-20, pH 7.4) for 1 hour and then incubated with anti-POC5 antibody in PBST with 5% BSA in PBT at 4°C ON. The secondary antibody was (anti-rabbit IgG secondary antibody (Thermoscientific cat: 31460) diluted at 1: 10000 for one hr at RT. In order to validate the mass spectroscopy data, and confirm the interaction of several ciliary proteins with POC5, Western blot was performed by stripping the same membrane with a Restore stripping buffer (Thermofisher cat: 21059) and probing it with different antibodies. The following antibodies were used Annexin2 (sc-374394), Galectin 3 and 7 (sc-32790 and sc-137085), CKAP5 (sc-374394), Desmocollin1 (sc-398590), CEP290 (ab84870), Septin9 (sc-293291), Acetylated-α**-**tubulin (Sigma Aldrich cat# T7451 1/2000), RAB11 (ab3612), EHD4 (sc-376373), Annexin5 (sc-32321), and CystatinA (sc-376759). All antibodies acquired from Santa Cruz (Sc) were mouse monoclonal and used at a dilution of 1/500. Rabbit polyclonal antibodies CEP290 and Rab11 were used at a dilution of 1/250. The secondary antibody (anti-rabbit IgG secondary antibody (Thermoscientific cat: 31460) was diluted 1:10000, and anti-mouse IgG secondary antibody (Thermoscientific cat: 31430) was diluted 1:10000 for 1 hour at RT. Membranes were exposed to ECL prime Western blotting detection reagent (GE healthcare cat: RPN2232) for 5 minutes at RT room.

### Immunoprecipitation phosphatase assay

HEK293 cells were transfected with wtPOC5 myc tagged or *POC5*^*A429V*^ myc tagged vectors (as described above) and then were synchronized at G1 or S phases. To block cells at G1 phase, cells were serum starved for 24 hours. To block cells at S phase, cells were first starved for 24 hours and then complete medium with serum (10% FBS) was added for 24 hours. For immunoprecipitation-phosphatase assays, total lysates were incubated with 2.5 μg anti-myc antibody as described above. Beads were blocked with 1% BSA and then washed three times and resuspended in 100 μl PBS. Then, protein antibody complex were incubated with beads. Alkaline phosphatase (NEB cat: M0290S) was added to the mixture and incubated at 37°C for 1 hour. Western blot was performed using POC5 antibody (Abcam cat: 188330) as described above.

### Quantification and statistical analysis

The length of cilia was quantified using ImageJ by measuring the acetylated-α-tubulin signals [20]. For ciliation experiments with NOB and AIS cells, approximatly 300 cilia were counted per experiment. For cell cycle analysis, the number of cells in S phase and G1 phase were counted. The percentage of cells in each phase over the number of total cells was determined. Approximately 100 cells were counted per phase. Mean values of individual experiments were plotted in bar graphs with ±SD between the individual sets. P-values were calculated by one-way analysis of variance (ANOVA). P<0.05 considered statistically significant.

## Results

### Altered subcellular localization of POC5 upon mutation

We first examined the subcellular distribution of wild-type human *POC5* (wt*POC5*) and an AIS-related mutant *POC5*^*A429V*^ (mut*POC5*) in HeLa cells. HeLa cells are p53-defective cell lines and hence they do not arrest at G1 phase after the disruption of centrosomal proteins, unlike non-transformed cells that are arrested at G1 through p53 dependent pathway upon depletion of centrosomal proteins. The centriolar cycle of HeLa cells is well described [21, 22]. For HeLa cell to progress through its life cycle, centriolar replication and pericentriolar changes occurs in synchrony with DNA synthesis and mitosis. Since POC5 localization and function has been well characterized in HeLa cells [7], we sought to first investigate the cellular effects of POC5 mutations in the HeLA cells.

**Fig 1:**
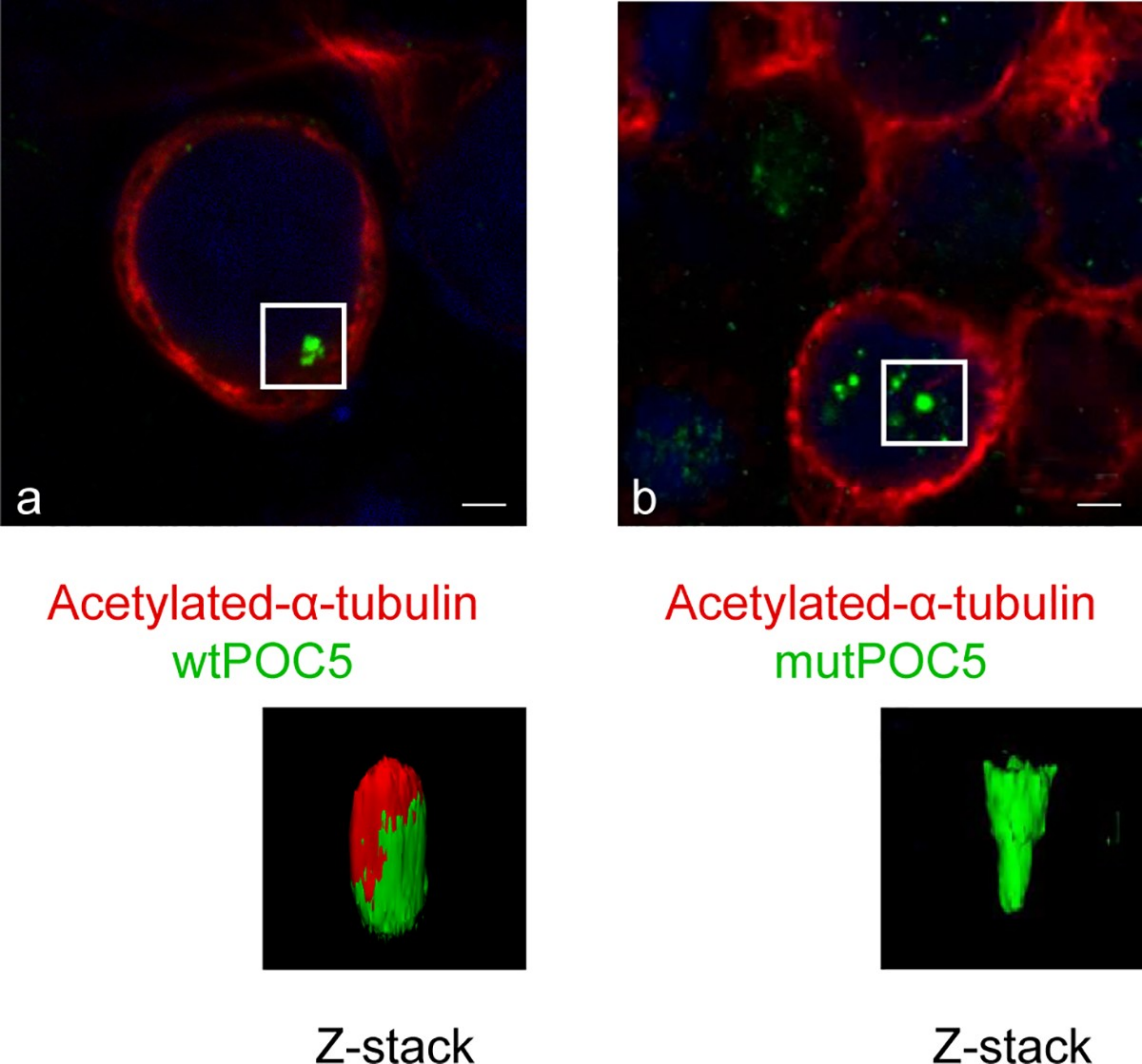
Differential localization of POC5 with acetylated-α-tubulin in wt*POC5* and *POC5*^*A429V*^ expressing cells. Confocal imaging of HeLa cells transiently transfected with wt*POC5* (a) or *POC5*^*A429V*^ (b). POC5 and acetylated-α-tubulin were determined using specific antibodies and positive signal revealed by green and red coloration respectively. Localization of POC5 with respect to acetylated-α-tubulin is shown: in cells transfected with wtPOC5 (a) where positive immunostaining was enriched at the perinuclear acetylated-α-tubulin ring, while in cells expressing *POC5*^*A429V*^ (b), POC5 was visualized inside the nucleus (disconnected from the perinuclear acetylated-α-tubulin ring). The Z-stack imaging (recording images at different focal planes) allows the visualization of the three-dimensional structure containing both POC5 and acetylated-α-tubulin (green and red) for the wtPOC5, while only POC5 (green) was visualized for the *POC5*^*A429V*^. Images were taken using the Zeiss microscopy Mag x40. Scale bar 1.6 μm. POC5 in green, acetylated-α-tubulin in red and DNA was stained with DAPI (blue).

In wt *POC5* cells, POC5 was found to localize to the acetylated-α-tubulin ring (Fig 1A). On the other hand, POC5 was mislocalized in *POC5*^*A429V*^ cells (Fig 1B).

### Aberrant POC5 localization in osteoblasts derived from AIS patients bearing the *POC5*^*A429V*^ mutation

**Fig 2:**
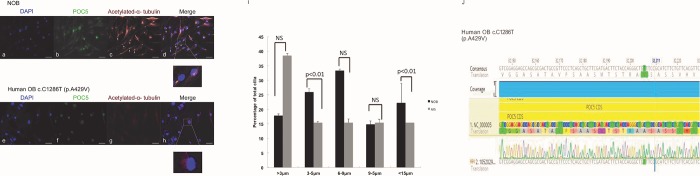
Human osteoblasts carrying *POC5*^*A429V*^ show short cilia. Representative immunodetection of POC5 and acetylated-α-tubulin by immunofluorescence in osteoblasts from normal (non scoliotic) (a, b, c, d) and cells with the *POC5*^*A429V*^ (e, f, g, h). Merged images (d and h) show differential colocalization of POC5 with respect to cilia. Zoomed image shows that POC5 is located at the cilium level marked by acetylated-α-tubulin. Human osteoblasts carrying the variant *POC5*^*A429V*^ (e, f, g, h), show decreased staining intensity and absence or retraction of cilium (h). *POC5*^*A429V*^ protein was seen to be localized within the nucleus (zoomed image). POC5 (green coloration), acetylated-α-tubulin (red coloration) and the nucleus were stained with DAPI (blue). Images were taken using the Zeiss microscopy. Mag x 40. Scale bar 1.6 μm. (I) The graphs represent the percentage of cilia for each length category in NOB and AIS cells (*POC5*^*A429V)*^. Each bar represents the mean of three independent experiments (±SD). P< 0.05 considered statistically significant. (J) Sequence alignment with Sanger sequencing confirm AIS cells to have the *POC5*^*A429V*^ mutation.

To confirm our observation in HeLa cells, we next investigated the effects of *POC5* mutation on the subcellular localization by comparing normal (non-scoliotic) to osteoblasts derived from AIS patients carrying the *POC5*^*A429V*^ variant (Fig 2). In normal control osteoblasts, POC5 expression was colocalized with acetylated-α-tubulin stainings, suggesting that centriolar protein POC5 is located at the cilium (Fig 2A–2D). In AIS osteoblasts, POC5 was rather located within the nucleus and not colocalized with acetylated-α-tubulin stainings (Fig 2E–2H). Furthermore, a retraction of cilium was observed in *POC5*^*A429V*^ osteoblasts (Fig 2H) compared to controls. More precisely, quantification of the ciliary length showed that *POC5*^*A429V*^ cells predominantly had shorter cilium of more than 3μm than that of control cells (Fig 2I). Altogether, these results suggest that mutations in POC5 affect its subcellular localization with respect to the cilium.

### Defects in cell-cycle progression in cells overexpressing the mut*POC5*

**Fig 3:**
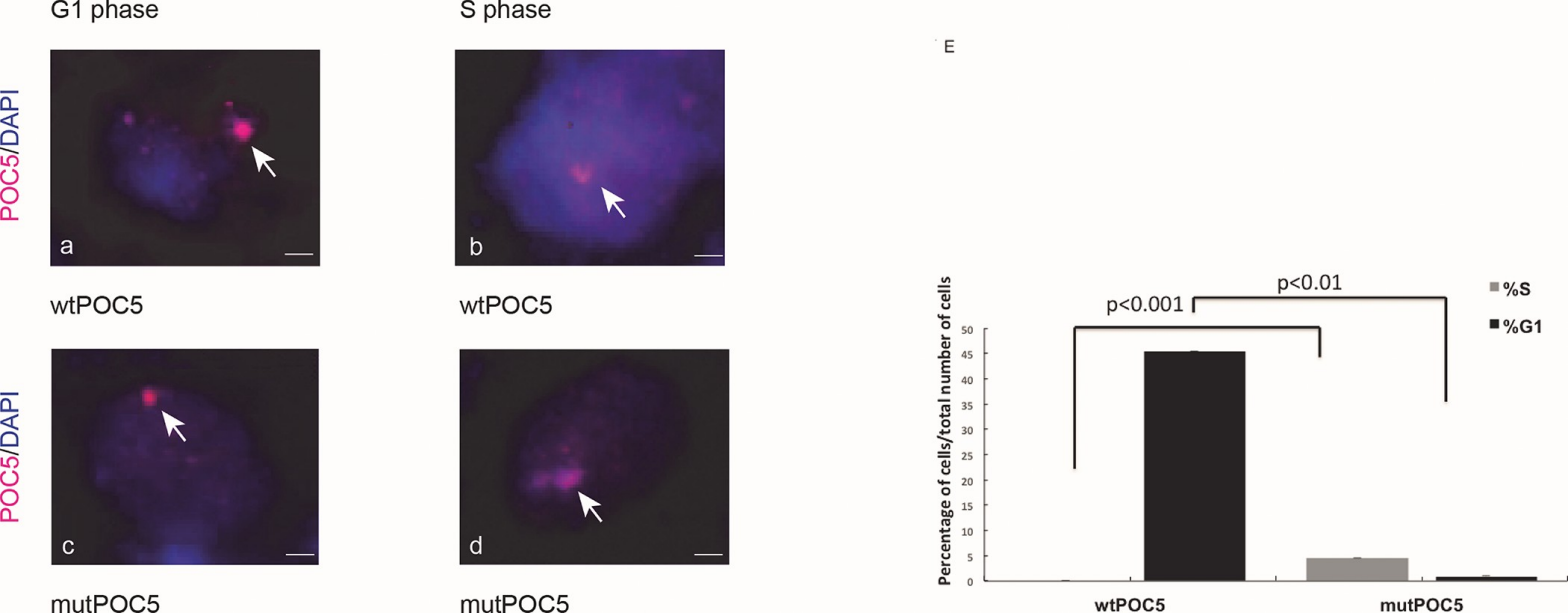
*POC5*^*A429V*^ expressing cells are arrested in S phase. Confocal imaging of HeLa cells overexpressing wt*POC5* or *POC5*^*A429V*^ specific staining for POC5 was performed using POC5 antibody. During G1 phase, obtained by serum starvation (a), wtPOC5 is located within the cytoplasm, while in the S phase (b) (serum replacement after deprivation), POC5 is located within the nucleus. WtPOC5 expressing cells have normal progression through G1 and S phases, (a, b). In the cells overexpressing *POC5*^*A429V*^, POC5 is located within the nucleus in both G1 and S phases (c, d). Cells are blocked in S phase and unable to progress through the cell cycle. E) The graphs represent the percentage of cells in G1 of S phase. Almost all wtPOC5 expressing cells are in G1 phase (45%), but *POC5*^*A429V*^ cells are accumulating in S phase. Each bar represents the mean of three independent experiments (±SD). P<0.05 considered statistically significant. Images were taken using the Zeiss microscopy. POC5 (visualized in red), DNA was stained with DAPI (blue). Mag x 40. Scale bar 1.6 μm.

**Fig 4:**
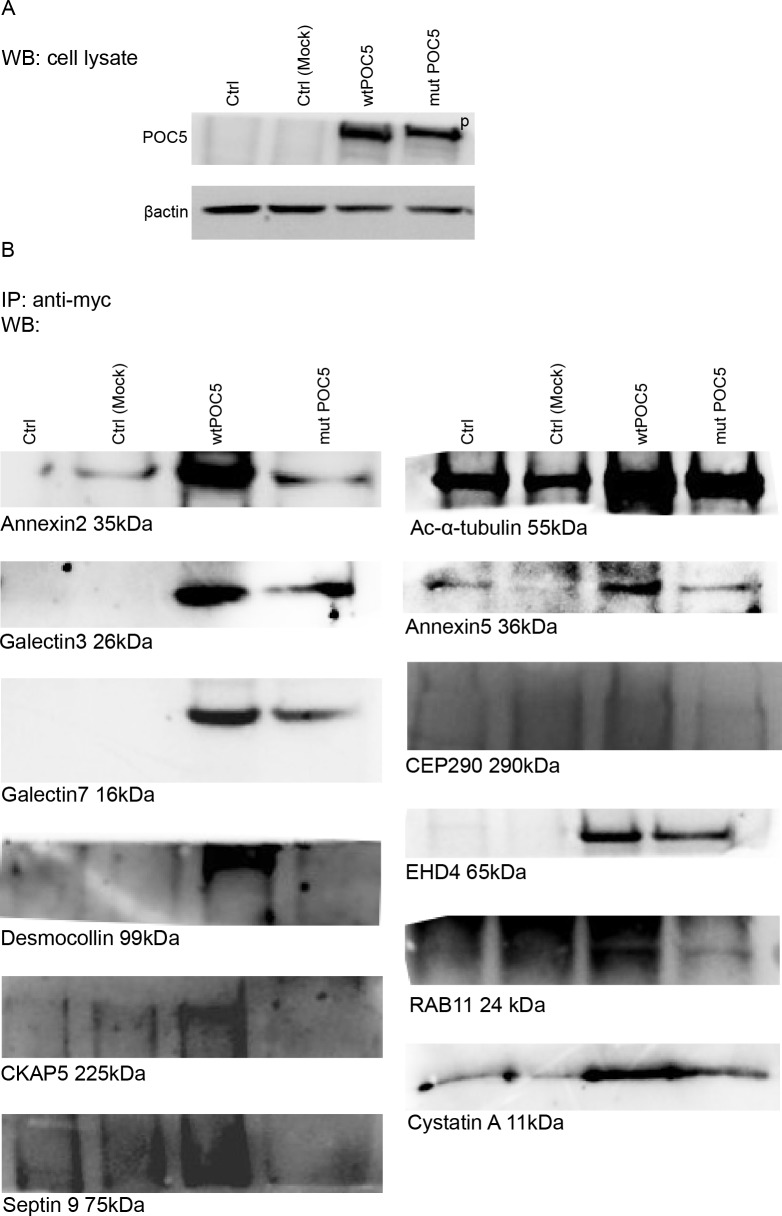
Validation of mass spectrometric results. Proteins identified by mass spectroscopy interacting with the wtPOC5 were analysed by CO-IP. Coimmunoprecipitation of myc-POC5 using anti-Myc antibodies in Hek293 cells. Proteins in the immune complexes were revealed by Western blotting with different antibodies as described in materials and methods. Control (cells transfected with Mock pCMV-entry vector) was used along cells transfected with myc tagged wt or *POC5*^*A429V*^ expressing cells. A) Western blot on total cell lysate using POC5 antibody. Wt and *POC5*^*A429V*^ tranfected cells have same expression levels of POC5. Mock transfected sample has very low expression of POC5. POC5 is observed at the expected size 63kDa in the wtPOC5 and at higher level in *POC5*^*A429V*^ (indicated as p: phosphorylated). B) CO-IP shows the binding of Annexin2, Annexin 5, Desmocollin1 and CEP290, exclusively with wtPOC5. Very weak interaction was observed between POC5^A429^ with Acetylated-α-tubulin, Galectin3 and 7, EHD4, CystatinA compared to wtPOC5.

Because POC5 is localized to the distal portion of centrioles and is recruited to procentrioles for full centriolar maturation and normal cell-cycle progression, we next determined how *POC5*^*A429V*^ protein is localized during cell cycle progression. HeLa cells transfected with either wt or *POC5*^*A429V*^ were synchronized in the growth phase (G1) cell cycle phase and then stained for POC5. In cells transfected with wt*POC5*, POC5 protein was observed outside the nucleus during the G1 phase (Fig 3A) and in the nucleus during the Synthesis (S)-phase (Fig 4B). However, in *POC5*^*A429V*^ expressing cells, POC5 was found to be rather localized in the nucleus through the G1 and S phases (Fig 3C and 3D). Interestingly, a significant increase (4%) in the proportion of S-phase cells was detected in *POC5*^*A429V*^ cells compared to wtPOC5 cells (Fig 3E). To confirm this observation, we performed cellular fractionation of cytoplasmic and nuclear extracts of cells overexpressing wt or *POC5*^*A429V*^. The wtPOC5 was concentrated in both nuclear and cytoplasmic fractions with higher expression levels in the cytoplasm. Contrary to wtPOC5, the *POC5*^*A429V*^ was exclusively found in the nucleus (Fig 4).

In order to check if the differential recruitment of wtPOC5 and *POC5*^*A429V*^ variant to the cilium is phosphorylation-dependent, we studied the pattern of phosphorylation of wt and *POC5*^*A429V*^ during the cell cycle progression. We found that the *POC5*^*A429V*^ was hyper phosphorylated independently of the phase of the cell cycle. In all cell cycle phases, the *POC5*^*A429V*^ protein had lower migration level than wtPOC5 protein and treatment with alkaline phosphatase shifted back *POC5*^*A429V*^ to an apparent molecular weight similar to wtPOC5 levels (S2 Fig).

### POC5 interacting protein partners are mostly ciliary and cytoskeletal proteins

**Fig 5:**
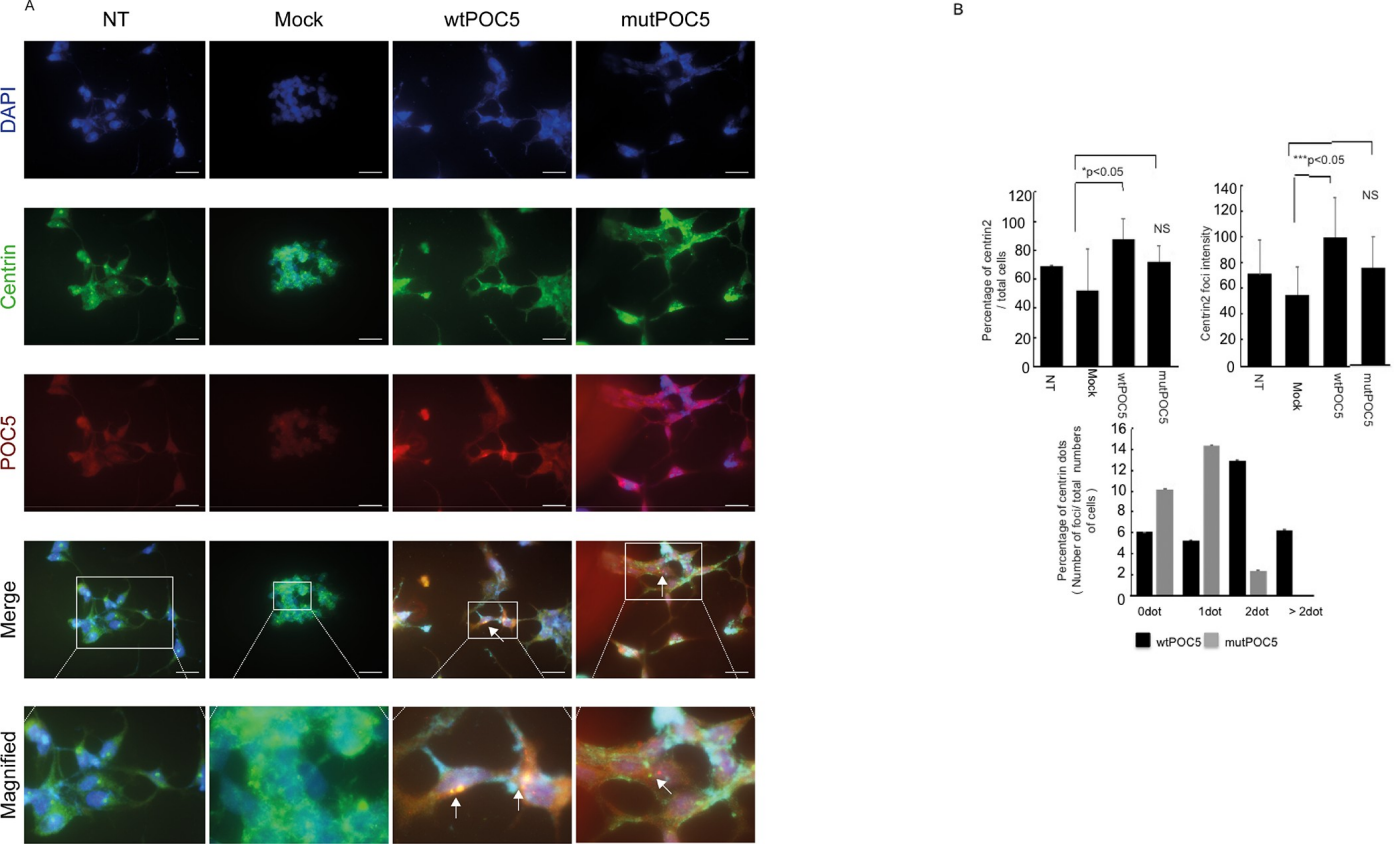
Differential colocalization of wtPOC5 and *POC5*^*A429V*^ with respect to centrin. A) Immunoflourescence staining was performed in HeLa cells non-transfected (NT) overexpressing pCMV-entry empty vector (mock), wtPOC5 or *POC5*^*A429V*^. Staining of POC5 (red), centrin 2 (green) shows colocalization of wtPOC5 and centrin 2 (merge orange colour), and absence of colocalization with *POC5*^*A429V*^ and centrin 2. B) Statistical analysis of centrin 2 expression, intensity and number of centrin foci per cell in mock, wt*POC5* and *POC5*^*A429V*^ expressing HeLa cells. Percentage of centrin 2 number and intensity are reduced in *POC5*^*A429V*^ expressing cells. Unlike wtPOC5 cells, most *POC5*^*A429V*^ cells have one foci of centrin but majority of wtPOC5 expressing cells have two foci of centrin 2. Centrin count and intensity was performed using ZEN software. Images (n = 6) were used for quantification. Error bars are the mean ± SD. p<0.05 considered statistically significant. POC5 (red), centrin (green) and DAPI (blue). NT: not transfected. NS: not significant.

To gain a better understanding of the centrosome and cilium protein interaction landscape in AIS, mass spectrometry studies were conducted in cells transfected with either the wt*POC5* or *POC5*^*A429V*^ cells following myc-tagged POC5 pull-down assays. Immunoprecipitation of POC5 showed similar expression levels of POC5 in both wt and *POC5*^*A429V*^ transfected samples (Fig 5A). We identified 85 proteins that interact predominantly with wtPOC5 but not *POC5*^*A429V*^ (Table 1) and only 5 proteins interacting with *POC5*^*A429V*^ (Table 2). Amongst these proteins, we identified 12 ciliary and cytoskeletal proteins that have altered interaction with POC5 when mutated. For instance, Annexin2, Desmocollin1 and Centrosomal protein 290kDa (CEP290) were found to interact with wtPOC5, but not with *POC5*^*A429V*^ (Fig 5B). Weaker interactions between POC5 and Galectin 3, Galectin 7, Acetylated-α-tubulin, EH domain-containing protein 4 **(**EHD4), Annexin5 and Cystatin A were observed in cells expressing *POC5*^*A429V*^ compared to wtPOC5 (Fig 5B and S1B Fig). Protein disulfide-isomerase A4, iduronate 2-sulfatase, golgi resident protein GCP60, aminopeptidase B, cDNA FLJ53442—highly similar to poly (ADP-ribose) polymerase 1 were found a new interactors of POC5 upon mutation (Table 2). These findings suggest that an altered centrosomal and cilia protein interaction may be involved in AIS.

**Table 1:** Mass spectroscopy results of proteins interacting exclusively with wtPOC5. Scaffold software was used for the analysis of the identified proteins interacting with wtPOC5. Protein identification in wtPOC5 expressing cells, detected 85 candidates interacting with wtPOC5. Clustering proteins by biological function indicated: ciliary proteins (17 proteins); cell adhesion (7 proteins); cytoskeleton-associated protein (4 proteins); RNA processing (9 proteins); extracellular matrix (3 proteins); response to estrogen (1 protein); cell cycle and cytokinesis (1 protein). Most of the identified proteins interacting with POC5 are cytoskeletal proteins (As shown in the table) and those marked in bold were considered for further analysis. Other protein groups belong to cell cycle and cytokinesis, extracellular proteins, RNA processing, cell adhesion, and response to estrogen.

**Protein ID**	**Description**	**Score**	**Peptide Number**
P15924	Desmoplakin	3984	119
Q08554	**Desmocollin-1**	581	12
P68363	**Tubulin alpha-1B chain**	368	10
P07355	**Annexin A2**	221	9
Q9NSK0-3	Isoform 3 of Kinesin light chain 4	155	9
F8VW92	Tubulin beta chain	251	7
P47929	**Galectin-7**	338	6
Q9H223	**EH domain-containing protein 41**	62	4
Q6IB90	**Cystatin**	84	3
Q9NZT1	Calmodulin-like protein 5	243	3
Q14574-2	Isoform 3B of Desmocollin-3	63	3
Q14008	**Cytoskeleton-associated protein 5**	57	3
Q9Y5P4-2	Isoform 2 of Collagen type IV alpha-3-binding protein	45	2
P27482	Calmodulin-like protein 3	144	2
P08758	**Annexin A5**	164	2
Q08380	Galectin-3-binding protein	76	2
**Protein ID**	**Description**	**Score**	**Peptide Number**
B4DF70	cDNA FLJ60461; highly similar to Peroxiredoxin-2 (EC 1.11.1.15)	88	2
Q59FR8	**Galectin 3**		2
Q9UHD8-5	**Isoform 5 of Septin-9**	46	2
J3KNF5	**Centrosomal protein of 290 kDa**	20	1
Q8N6N5	Tubulin; beta 2C	85	1
B4E0R6	Importin-5	69	1
Q86YS3-2	**Isoform 2 of Rab11 family-interacting protein 4**	27	1
A8MUB1	**Tubulin alpha-4A chain**	183	1
A9X9K9	Desmocollin 2	46	1
r-Q5T802	RUNX2	15	1
Q13835	Plakophilin-1 2	536	17
F5GWP8	Junction plakoglobin	3102	16
P25311	Zinc-alpha-2-glycoprotein	141	3
Q9GZZ8	Extracellular glycoprotein lacritin	98	3
E9PBV3	Suprabasin	190	3
Q9Y5P4-2	Isoform 2 of Collagen type IV alpha-3-binding protein	45	2
B4DT31	Far upstream element-binding protein 1	151	9
E9PIN3	Nuclear RNA export factor 1 (Fragment)	149	5
O43776	Asparagine—tRNA ligase; cytoplasmic	94	4
Q9Y2X3	Nucleolar protein 58	113	4
B8ZZD1	U4/U6.U5 tri-snRNP-associated protein 2	43	2
P35579	Myosin-9	555	18
P58107	Epiplakin	200	10
Q8WVV4	Protein POF1B	242	6
Q15149-9	Isoform 9 of Plectin	84	2

**Table 2:** Mass spectroscopy results of proteins interacting exclusively with POC5^A429V^. Five proteins were found to be interacting exclusively with POC5^A429^. Protein disulfide-isomerase A4, Iduronate 2-sulfatase, Golgi resident protein GCP60, Aminopeptidase B and cDNA FLJ53442; highly similar to Poly (ADP-ribose) polymerase.

Protein ID	Description	Score	Peptide Number
P13667	Protein disulfide-isomerase A4	226	9
P22304	Iduronate 2-sulfatase	138	5
Q9H3P7	Golgi resident protein GCP60	193	3
Q7RU04	Aminopeptidase B	95	2
B4E0E1	cDNA FLJ53442; highly similar to Poly (ADP-ribose) polymerase 1	61	2

### WtPOC5 but not *POC5*^*A429V*^ colocalizes with centrin

**Fig 6:**
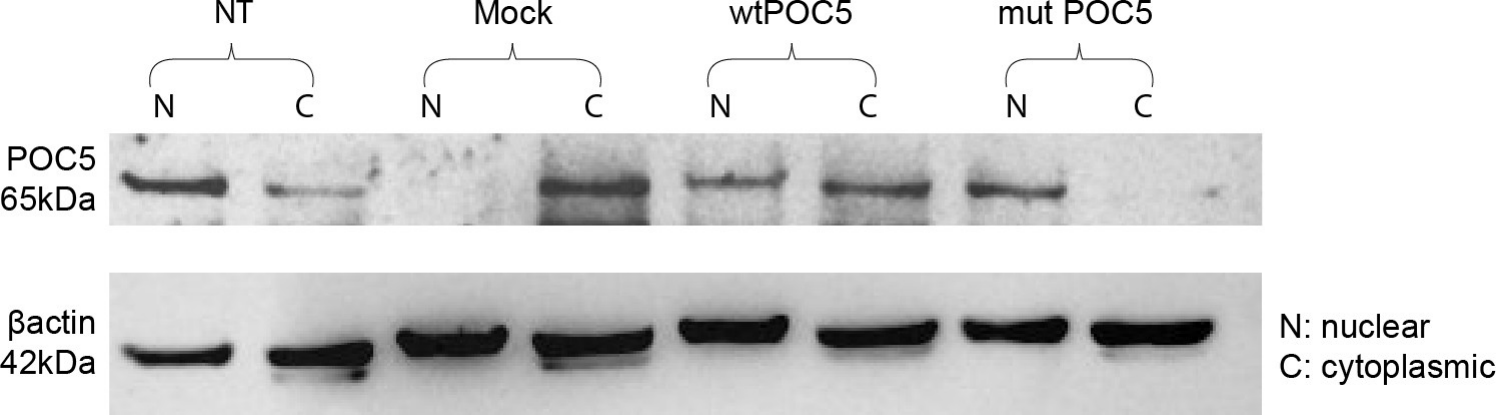
Differential subcellular localization of wtPOC5 and *POC5*^*A429V*^. Nuclear and cytoplasmic cell extracts were obtained using protocol as described in materials and methods. Equal nuclear and cytoplasmic protein samples loaded were determined using β-actin as loading control and subjected to immunoblotting using anti-POC5 antibody. Controls were cells non transfected or transfected with pCMV-entry vector. The wtPOC5 is mainly expressed in the cytoplasm and the *POC5*^*A429V*^ is mostly nuclear. The results are from two independent experiments (n = 2).

Localization of hPOC5 and centrin during the cell cycle has previously been described in literature [7]. To precisely localize POC5 and to study the consequence of mutation on POC5 recruitment to cilia, we performed double immunostaining for POC5 and centrin in cells overexpressing wtPOC5 or *POC5*^*A429V*^. In immunofluorescence microscopy, anti-hPOC5 antibody staining labeled two foci for wtPOC5 and one foci for *POC5*^*A429V*^. These foci represent centrioles, as confirmed by colocalization with human centrin (Fig 6A). Quantification of centrin percentage per cell revealed that there is significantly higher centrin staining intensity in wtPOC5 expressing cells than *POC5*^*A429V*^ cells and that the *POC5*^*A429V*^ expressing cells had mainly one or more than two centrin foci compared to wtPOC5 cells which had predominantly 2 foci (Fig 6B).

## Discussion

**Fig 7:**
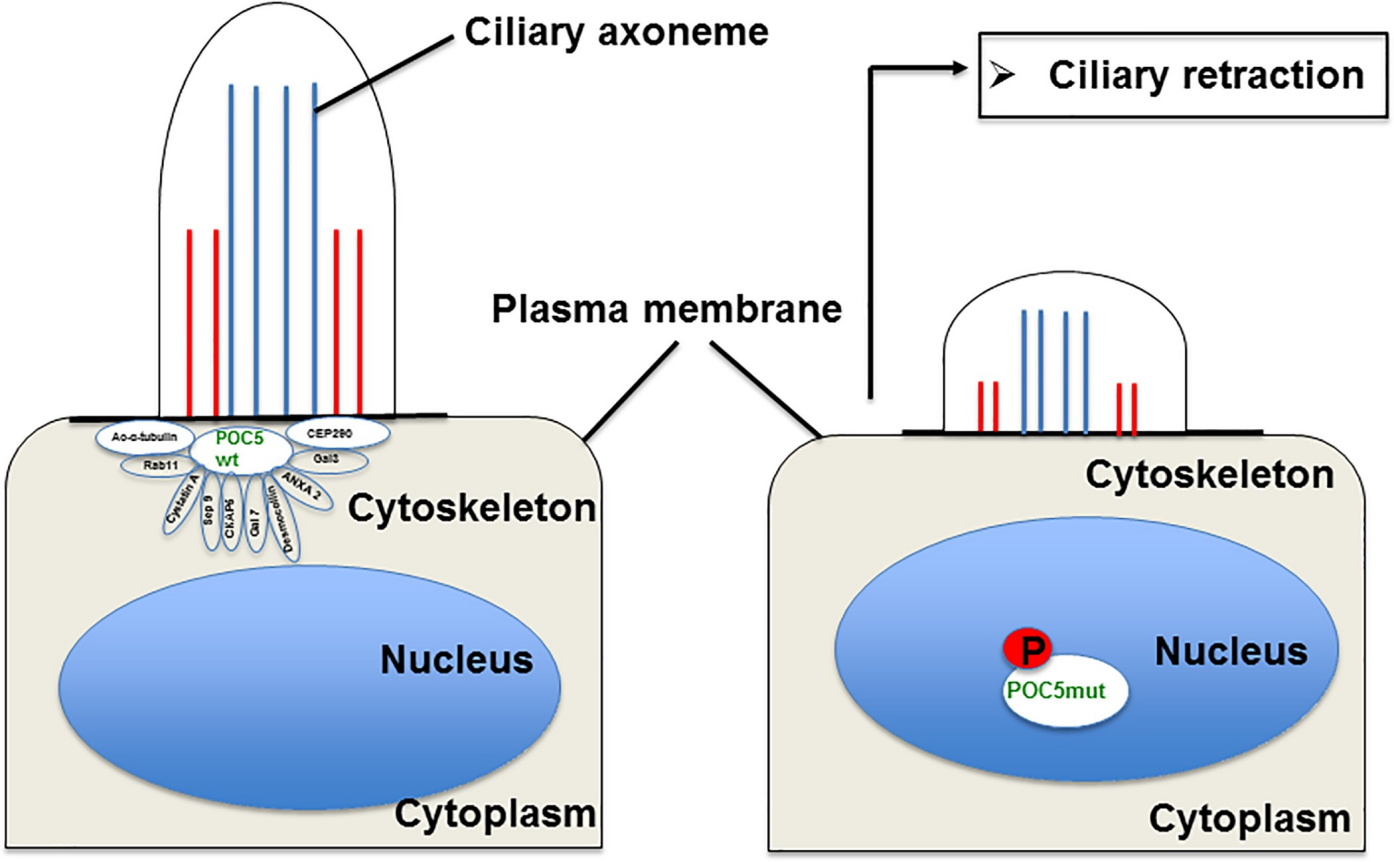
Proposed model for the mechanisms of ciliary retraction in *POC5*^*A429V*^ expressing cells. Under normal conditions, POC5 is an essential protein for normal cell cycle progression, and this process is a tightly regulated mechanism. In cycling cells, with wtPOC5, POC5 protein is found interacting with several ciliary proteins that assemble before entering to G1 phase. Weak or no interaction of *POC5*^*A429V*^ with ciliary proteins (revealed in this study by immunofluorescence, mass spectroscopy, and CO-IP) results in incorrect assembly and cilium retraction.

In this study, we demonstrated that the mutation in POC5 ^A429V^, previously described in patients with AIS [5], resulted in an altered subcellular localization of POC5 both in transfected and clinically-relevant patients cells. We also showed that several cilia, microtubules, cytoskeleton, and centrosomal proteins interact with POC5 and many of which are lost with an AIS-related POC5 mutation. We further found an impaired cell cycle progression and ciliary retraction in cells expressing POC5^A429V^ compared to controls (Fig 7).

In French families with AIS, various functional variants (c.G1336A (p.A446T), c.G1363C (p. A455P), and c.C1286T (p.A429V)) are contributing to the occurrence of AIS [5]. Recently, a common variant of *POC5* was associated with the susceptibility to AIS (single-nucleotide polymorphism (SNP) rs6892146)) in Chinese population [6]. This common SNP has a significantly different distribution of minor allele frequency in patients with the GG and with CC genotype. Interestingly, in patients with the GG genotype, POC5 mRNA expression was found to be significantly increased when compared to the controls [6]; however, little is known about the function of POC5 and its role in AIS pathologies. The *POC5* variant, *POC5*^*A429V*^ is a rare variant that was found in AIS patients [5].

In addition to the association of POC5 with AIS [5], recently, new mutations in *POC5* gene were associated with Retinitis Pigmentosa (RP) [10]. Retinitis Pigmentosa is a photoreceptor degenerative disease, characterized by the degeneration of rod and cone photoreceptors and is classified as ciliopathy disease. Weisz et al [10] reported POC5 to be localized at the connection cilia in the photoreceptors, and when mutated, the length of photoreceptor outer segments is reduced. In our *in vitro* work, in cells overexpressing *POC5*^*A429V*^, POC5 was not co-localized with the acetylated-α-tubulin (Fig 1B), dispatched from centrin (Fig 5) and solemnly localized in the nucleus (Fig 6). In contrast, the wtPOC5 was found to be interacting with several ciliary proteins (Table 1). We point out to a role of POC5 as a ciliary protein that is located at the base of the cilium as was confirmed by centrin staining (Fig 5).

Our immunofluorescence results showed also a differential localization of wt and *POC5*^*A429V*^ with respect to the centrin (Fig 5). Our mass spectroscopy and co-IP data (Fig 4) showed several ciliary proteins to be interacting exclusively with wtPOC5 but not *POC5*^*A429V*^. Among these two proteins component of cilia were found; RAB11 and CEP290. Protein RAB11 is part of the multiple GTPases group that include RAB6, RAB11, and RAB8A which are involved in the trafficking to the cilium. Interestingly, several disorders associated with genetic mutations encoding defective proteins of cilia formation or function showed scoliosis as a secondary manifestation. Some studies have shown that the potential handover mechanisms may exist between RAB11 and RAB8 at the base of the cilium [23]. Rab8 and Rab11 were found to be associated with the Bardet-Beidl syndrome (BBS) pathway [24]. CEP290 is also known to be an important component of the primary cilium, localizing to the Y-links of the ciliary transition zone and having a role in the regulation of transport in and out of the ciliary compartment [25]. Furthermore, CEP290 mutations lead to a range of ciliopathy syndromes with variable clinical manifestations in humans: BBS, Joubert syndrome and Meckel-Gruber syndrome [26]. Given that both proteins (POC5 and CEP290) are located at the base of the cilium (as also confirmed in this study by centrin immunostaining), and that CEP290 interacts solely with wtPOC5, this supports the role of POC5 as a ciliary protein operating at the base of the cilium.

Interestingly, two proteins previously associated with scoliosis, Annexin A2 and Calmodulin were found to interact with wtPOC5 were previously associated with scoliosis. Annexin A2, is highly expressed in osteoblasts, and differentiated growth plate chondrocytes and plays an essential role in bone mineralization which appears to be critical in AIS patients. Calmodulin-like protein 3 (immunoprecipitated with wtPOC5 but not *POC5*^*A429V*^) is another interesting putative protein partner of POC5 in AIS. Calmodulin-like protein is a calcium sensor protein that is closely related to the ubiquitous calmodulin, which is considered potential key molecule in the etiology of scoliosis because of its effects on muscle contractility, that was reported defective in AIS pateients [27] (Table 1). Only five proteins were found to be associated with mutPOC5 protein 5. Interestingly, among those proteins is iduronate 2-sulfatase. Iduronate 2-sulfatase is associated with Hunter syndrome clinical disorder (mucopolysaccharidosis type II, MPS-II) in which patients may display, scoliosis [28, 29]. Also, the cellular stress gene protein disulfide isomerase (PDIA4) was found to be associated with *POC5*^*A429V*^. PDIA4 is up-regulated in mouse models of brain neurodegenerative diseases involving protein misfolding. Although not much is known about the physiological role of PDIA4, studies indicate that this gene is upregulated following endoplasmic reticulum ER stress [30, 31]. Another interesting observation was the hyper-phosphorylation of the *POC5*^*A429V*^ (S2 Fig). It is well known that posttranslational modifications are one of the ways to regulate protein activity, subcellular localization, and stability [32]. Further studies are needed to determine where exactly the phosphate group is added and the accurate consequences on the physico-chemical properties, stability, kinetics, and dynamics [32].

One of the most intriguing results of our study was the immunofluorescence on human cells carrying the *POC5* variant (c.C1286T; p.A429V mutation) showing the subcellular mislocalization of POC5. We observed ciliary retraction in scoliotic osteoblasts (Fig 2H) with the *POC5*^*A429V*^ mutation (Fig 2H) when compared to the normal osteoblasts (Fig 2D). The primary cilium is an antenna-like projection of the cell that plays a critical role in the perception and integration of environmental signals like mechanotransduction. Cilia are also essential for left-right (L-R) symmetry during embryonic development [33, 34] and for cerebrospinal fluid (CSF) flow [35]. Motile cilia dysfunction may cause spinal deformity similar to the IS [17] and restoration of motile cilia activity stopped spinal curve progression, as evidenced recently in a zebrafish model [17]. This observation supports our results that show a defect in cilia organization as well as perturbed localization of POC5 with respect to the cilia in cells expressing a *POC5* mutation (Fig 2H). Another interesting observation is the defect in the cell cycle in *POC5*^*A429V*^ overexpressing cells. The cells were synchronized to be at the same cell cycle stage, and a different subcellular localization of wt and *POC5*^*A429V*^ at different stages of the cell cycle was observed. Not only the cellular localization of POC5 but also the number of centrin foci were used to confirm the accumulation of *POC5*^*A429V*^ in S phase (>2 foci of centrin) [7, 36]. The *POC5*^*A429V*^ was permanently located within the nucleus in G1 and S phases (Fig 3C and 3D). By cellular fractionation, we confirmed the different subcellular localization of wt and *POC5*^*A429*^ (Fig 6). Previous work [7] also showed that a depletion of POC5 in HeLa cells affects the progression through S phase. Human h-POC5-depleted cells had a significant increase in the proportion of S-phase cells; thus, hPOC5 depletion induced an accumulation of cells in S phase where procentriole assembly was probably initiated; however, these procentrioles failed to elongate. The cilia and neurosensory component of this pathology merits further investigation through various functional tests in patients with AIS in order to evaluate the possible functional defect connected with the altered structures identified in this work. Further study, focused on the primary cilia-mediated function, will provide more insight into the molecular mechanisms and etiology of AIS.

## Conclusion

This work identified specific protein interaction partners of POC5 and revealed that *POC5*^*A429V*^ alters the interaction with several ciliary and cytoskeletal proteins. Improved interaction of *POC5*^*A429V*^ with ciliary proteins resulted in the loss of POC5 co-localization with acetylated-α-tubulin, impaired cell cycle progression and cilium retraction These findings open new avenues for the understanding the role of POC5 in AIS at the molecular and cellular levels and suggest that centrosomes and cilia may underly AIS pathogenesis

## Supporting information

S1 Fig Acetylated-α-tubulin is highly enriched in precipitated wtPOC5 lysate but not *POC5*^*A429V*^. Hek293 cells were transfected with empty pCMV entry vector, wtPOC5 or *POC5*^*A429V*^ myc tagged vectors. Immunoprecipitation of POC5 was performed using myc antibody (origene). A) Western blot of acetylated-α-tubulin after immunoprecipitation of POC5, shows high expression of acetylated-α-tubulin in the wtPOC5 expressing sample and lower levels in *POC5*^*A429V*^. Very low levels are observed in mock transfected sample. B) Coommasie blue staining shows similar levels of POC5 expression in wt and *POC5*^*A429V*^ transfected samples. POC5 is observed at the expected size 63kDa. Also the heavy and light chains of antibody are observed.
(TIF)

S2 Fig Phosphorylation state of wtPOC5 and *POC5*^*A429V*^ at G1 and S phases of cell cycle. The immunoprecipitated samples were either non treated or treated with alkaline phosphatase and then western blot was performed using POC5 antibody (abcam). The presence or absence of phosphorylation with wtPOC5 and *POC5*^*A429V*^ is shown at G1 and S phase. POC5 wt is not phosphorylated, but the *POC5*^*A429V*^ is phosphorylated, and treatment with phosphatase dephosphorylates *POC5*^*A429V*^ that returns back to the same levels of wtPOC5. Phosphorylation of mutPOC5 is seen at both G1 and S phases.
(TIF)
