# Supplementary figures and images for: Adolescent idiopathic scoliosis associated POC5 mutation impairs cell cycle, cilia length and centrosome protein interactions
Source: PLoS One. 2019 Mar 7;14(3):e0213269. doi: 10.1371/journal.pone.0213269 (PMC6405090; doi:10.1371/journal.pone.0213269)

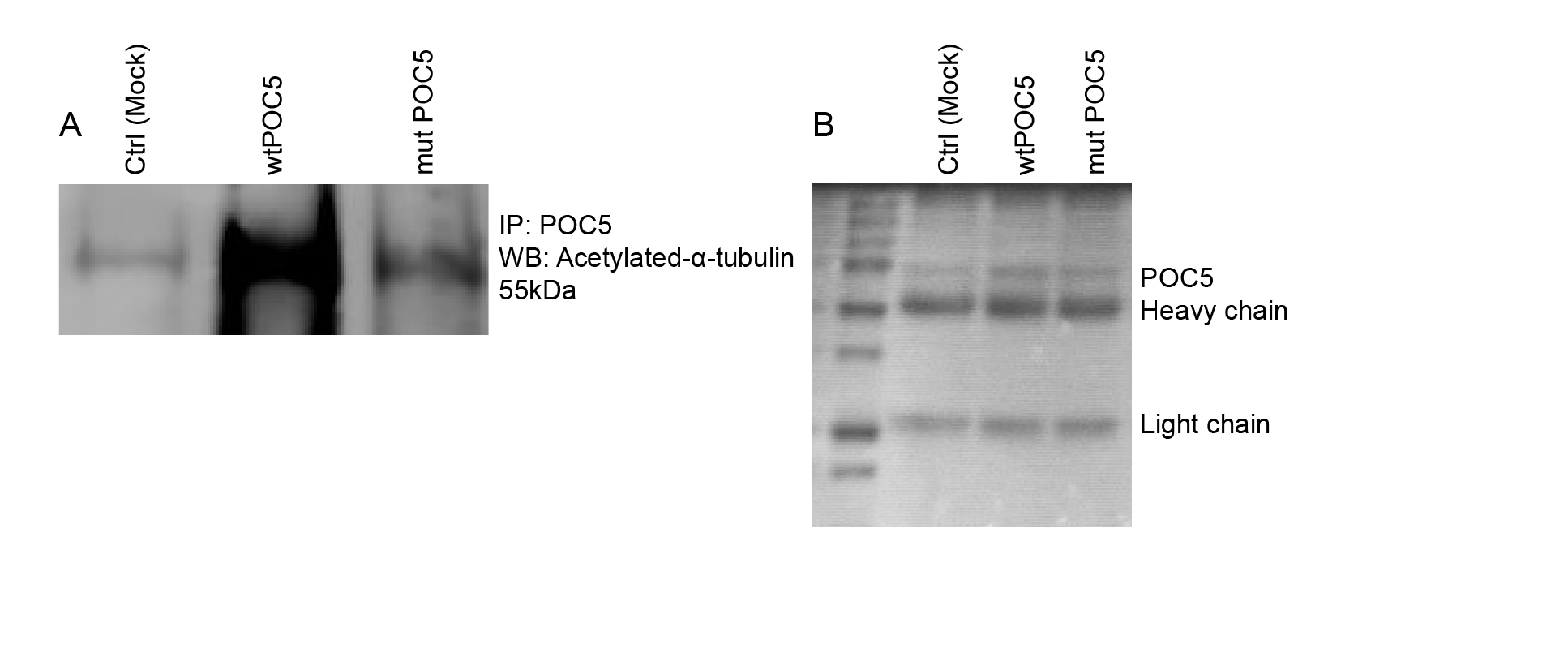

Supplement: S1 Fig — Hek293 cells were transfected with empty pCMV entry vector, wtPOC5 or POC5A429V myc tagged vectors. Immunoprecipitation of POC5 was performed using myc antibody (origene). A) Western blot of acetylated-α-tubulin after immunoprecipitation of POC5, shows high expression of acetylated-α-tubulin in the wtPOC5 expressing sample and lower levels in POC5A429V. Very low levels are observed in mock transfected sample. B) Coommasie blue staining shows similar levels of POC5 expression in wt and POC5A429V transfected samples. POC5 is observed at the expected size 63kDa. Also the heavy and light chains of antibody are observed. (TIF) [file pone.0213269.s001.tif]

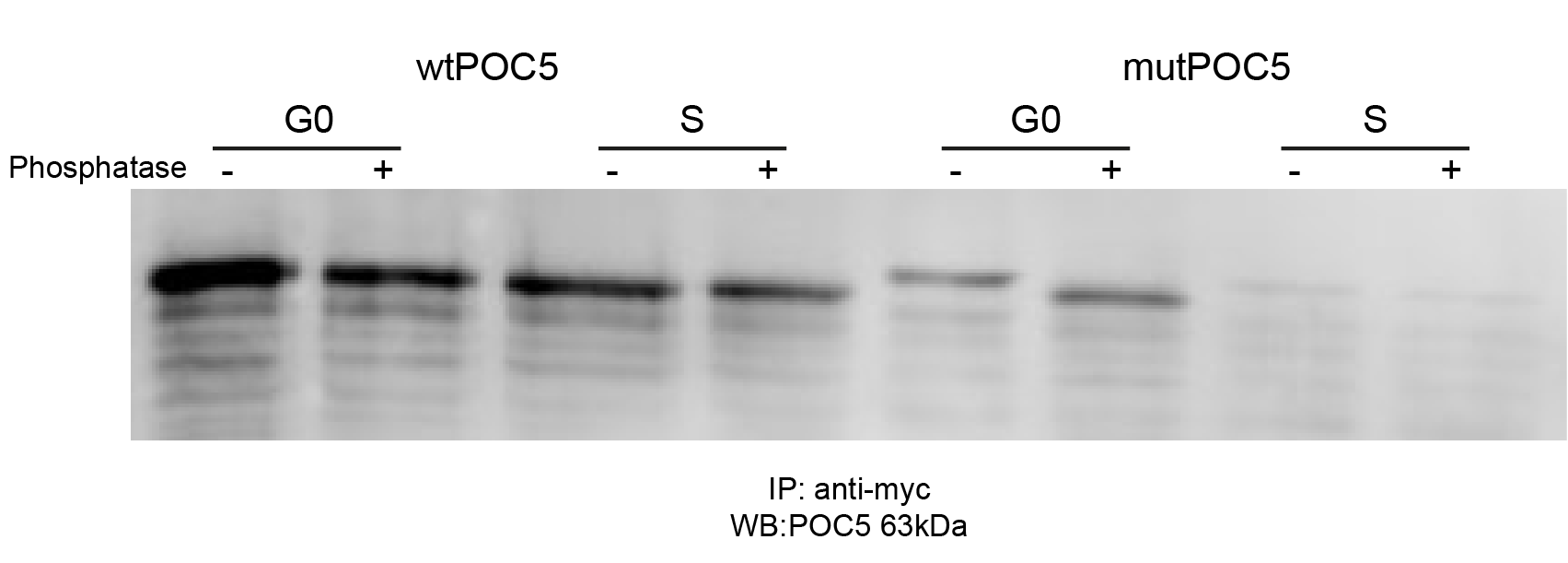

Supplement: S2 Fig — The immunoprecipitated samples were either non treated or treated with alkaline phosphatase and then western blot was performed using POC5 antibody (abcam). The presence or absence of phosphorylation with wtPOC5 and POC5A429V is shown at G1 and S phase. POC5 wt is not phosphorylated, but the POC5A429V is phosphorylated, and treatment with phosphatase dephosphorylates POC5A429V that returns back to the same levels of wtPOC5. Phosphorylation of mutPOC5 is seen at both G1 and S phases. (TIF) [file pone.0213269.s002.tif]
